# Senescent-associated β-galactosidase accumulation does not correlate with lifespan in different strains of *Nothobranchius furzeri* but does respond to intermittent fasting to extend lifespan

**DOI:** 10.17912/micropub.biology.001975

**Published:** 2026-05-28

**Authors:** Israel Akinrotimi Akinola, Tyrone Genade

**Affiliations:** 1 Department of Medical Education, East Tennessee State University, Johnson City, Tennessee, United States

## Abstract

Senescent cell (SC) accumulation theoretically causes aging-related tissue malfunction. We examined SC burden in short-lived and long-lived *Nothobranchius furzeri* fish strains, and fasting's effects on SCs. Despite different lifespans, SC accumulation rates didn't correspond between strains. The long-lived strain showed sex-based lifespan differences without SC burden differences. A 14-day fast reduced SC burden in short-lived fish, but this effect was temporary. Fasting extended median lifespan without affecting maximum lifespan, suggesting transient healthspan improvement. We conclude that saβgal-assessed SC burden doesn't necessarily correspond with lifespan between strains but may within strains. Our findings confirm intermittent fasting improves health and median lifespan.

**Figure 1. Lifespan curve and sa- β -galactosidase staining results for two N. furzeri strains (GRZ & MZM):** Figure 1: **(A & B)** Lifespan and cumulative hazard curves of the two *N. furzeri* strains: Gonarezhou (GRZ, n = 318) and MZM 04-3 (MZM, n = 153). MZM fish live longer than GRZ fish with median lifespans of 182 and 84 days respectively. **(C & D)** No difference in lifespan was found between the sexes for the GRZ strain (n = 58 male, 56 female) but **(E & F)** a significant difference of 45.4% was present for the MZM strain (n = 58 male, 40 female living 130 and 189 days respectively). **(G & H)** An early-effect lifespan difference was found between control (n = 295) and fasted (n = 68) GRZ fish. **(I)** A large strain and age effect in sa- *β* -galactosidase staining was found between the strains. **(J, K & M)** No difference was found between MZM age groups nor between 141-day old MZM males and females. **(L, N & O)** A large age and experiment effect was found for GRZ fish. Note the large difference in staining intensity between MZM and GRZ fish ( *η* ^2^ = 0.88) and the sharp decrease in staining intensity in fasted fish at 58 days of age (Cohen’s d = 5.2). Each dot represents an individual fish. Error bars are standard errors; *: p *<* 0.5; **: p *<* 0.1.

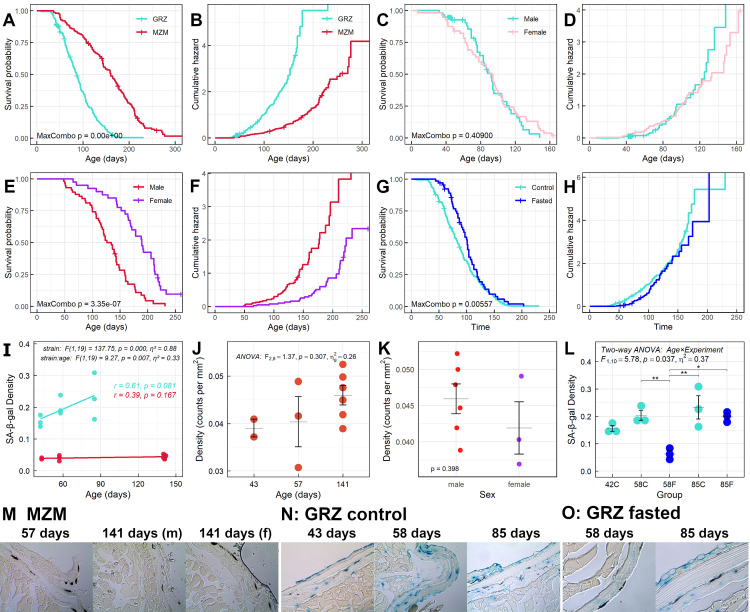



## Description

Senescent cells (SCs) accumulate with age where they cause tissue malfunction and disease that predisposes the organism to increased risk of mortality (Burton, 2009). The clearance of SCs is a critical aspect of tissue homeostasis and regeneration (Baar et al. 2017) and their removal has been shown to improve health and increase lifespan (Deursen, 2019). SCs are also predisposed to accumulate cellular waste, as evidenced by the accumulation of lysosomal *β* -galactosidase which causes the reported senescence-associated *β* -galactosidase (sa *β* gal) activity (Lee et al. 2006) – a hallmark of SCs.

Intermittent fasting has proven to be a reliable means of reducing age-related disease and increasing lifespan in a wide range of organisms (Cabo et al. 2019). Fasting is reported to have a variety of positive effects (Reddy et al. 2024), and intermittent fasting has been taken up as a dietary fad (Anton et al. 2018). However, the long-term effects are unknown – particularly in relation to effects on lifespan (Dong et al. 2024). There is evidence that intermittent fasting increases autophagy (Ehrnhoefer et al. 2018) and it is hypothesized that intermittent fasting stimulates apoptosis of SCs (Longo et al. 2014), but this has not been definitively demonstrated outside of the immune system (Cheng et al. 2014).

*Nothobranchius* constitute a powerful model with a median lifespan of 12 weeks (Cellerino et al. 2016, Wilcox et al. 2021, Terzibasi Tozzini et al. 2020). Previous experiments have demonstrated that *N. furzeri* responds to both dietary restriction (every other day feeding) with lifespan extension (Terzibasi et al. 2009). Additionally, a short treatment with senolytics reduced SC burden and increased neuroregeneration (Van Houcke et al. 2023). Many strains of *N. furzeri* are currently employed in research and there is evidence that these strains develop different tissue pathologies and age differently (Terzibasi et al. 2008; Wilcox et al. 2021).

*Nothobranchius* fish are reported to develop a senescence-associated *β* -galactosidase activity over time (e.g., Schöfer et al. 2024). If SCs underlie aging and lifespan, we hypothesize that short- and long-lived strains of *N. furzeri* will accumulate sa *β* gal stained cells at different rates and that the burden of sa *β* gal stained cells will correlate with lifespan. As senolytics extend lifespan and intermittent fasting extends lifespan we hypothesize that fasting will extend the maximal lifespan of *N. furzeri* as well as reduce the sa *β* gal staining.

To test the hypotheses that sa *β* gal stained cells increase at different rates in short- and long-lived strains, and that the sa *β* gal stained cell burden corresponds with lifespan, we studied the short-lived Gonarezhou (GRZ) strain and the long-lived strain MZM 04-3 (MZM) from Mozambique (Terzibasi et al. 2008). There are both large aging phenotype and genetic differences between these strains (Bartakova et al. 2013; Terzibasi et al. 2008). We chose to monitor SCs burden in skin because this methodology can most easily be adapted for non-lethal mammalian experiments to test the generality of the results generated from these experiments.

We confirm a large difference in lifespan between GRZ and MZM fish ( Figure 1 A & B). We show that there was no difference in lifespan between male and female GRZ, while there was for the MZM strain where females lived 45.4% longer than males ( Figure 1 A–F). In accordance with our hypothesis we expected that sa *β* gal levels would be similar at median lifespan even though the SC accumulate at a different rate. However, we show that there is a large difference in sa *β* gal density between the strains (Two-Way ANOVA, p = 0.0, *η* ^2^ = 0.88). What is more is that, while the sa *β* gal density trends up for the GRZ strain (Two-Way ANOVA, p = 0.007, *η* ^2^ = 0.33), there is little increase for MZM over time. A significant correlation between sa *β* gal and age was not found in either strain due to high variability in the data (coefficient of variation of 31.4% for the 85-day GRZ data and average coefficient of variation of 32.96% for the and MZM data as a whole). Additional sampling is required for the 85-day time-point (GRZ) and all MZM time points. Surprisingly, there was no difference in sa *β* gal density between the MZM sexes at 141-days of age ( Figure 1 E & F). Together this is evidence against our hypothesis that sa *β* gal burden corresponds with strain lifespan. Fish of the MZM strain are reported to accumulate levels of lipofuscin – another hallmark of aging and senescence associated with lysosomal dysfunction–with age in excess of the GRZ levels (Terzibasi et al. 2008). In *N. furzeri* lipofuscin content may therefore not directly correlate with sa *β* gal staining as in other models of aging (Bertolo et al. 2019; Flor et al. 2022) and supports the hypothesis that sa *β* gal and lipofuscin accumulation are symptoms of different aspects of lysosomal dysfunction (Ogrodnik et al. 2024).

To determine if intermittent fasting reduces the burden of senescent cells and increases lifespan we fasted individuals of the short-lived GRZ strain for 14 days from 42-days of age and assessed sa *β* gal staining at 58- and 85-days in fasted and control individuals. An early lifespan-extending effect was observed with mean lifespans of 85 vs 101 days ( Figure 1 G & H, p = 0.00557, MaxCombo test). The hazard rate of fasted fish was initially reduced during the fasting period but accelerated afterwards so as to match the control fish. A large decrease in sa *β* gal staining and cell counts were observed at 58-day old fasted fish compared to control (p *<* 0.01, Cohen’s d = 5.2) but for 85-day old fish there was no difference. No difference in lifespan between male and female fasted fish was found (p = 1, MaxCombo).

These findings imply that a fasting period has a strong effect on median lifespan but does not affect maximum lifespan. This indicates that intermittent fasting increases healthspan in *N. furzeri* GRZ. This result is in contradiction with our hypothesis that reducing sa *β* gal burden will increase maximum lifespan. This is in accordance with the data of Gavrilova et al. (2025) where an increasing morality rate at older ages is a trade-off for a decrease in mortality at younger ages. It is not clear what became of the senescent cells in the 42-day old fish once fasting began. It is hypothesized they undergo apoptosis (Cabo et al. 2019). If this is the case then the rapid return of senescent cells indicates new cells are becoming senescent at a faster rate than their predecessors such that SC burdens are again similar at 85-days of age. This could be evidence of stem cell aging, and that the cells descended from the aged stem cells themselves start their life at an advanced state of age. This is supported by the findings of Volodin et al. (2025) where regenerated fins of *N. guentheri* have the same expression profile as age-matched controls. An alternative interpretation is that the period of fasting induced a metabolic shift where cell-recycling, repair and protective enzymes were up-regulated - as reported in other studies (Longo et al. 2014; Ehrnhoefer et al. 2018) - and caused the clearance of accumulated *β* -galactosidase enzyme. In this scenario, on the resumption of feeding the damaged senescent cells were still there, and rapidly re-accumulated *β* -galactosidase enzyme. These hypotheses, or how much each may contribute to the observations, can be tested using the experimental system of Volodin et al. (2025) where a combination of cell labeling (with, e.g. EdU) can be used to track cellular turnover during aging and fasting as well as apoptosis rates.

While fasting experiments were not carried out in MZM fish, it would be useful to determine how the sa *β* gal staining changes under fasted conditions in this strain. Following up with western blotting of autophagic, cellular repair and defense enzymes will clarify what occurs during the fasting period, and provide insight into the observed aging differences between male and female MZM fish. It also needs to be determined how quickly the sa *β* gal stain intensity and number of sa *β* gal labeled cell density changes, what the optimal fasting duration might be and whether repeated periods of fasting can extend both health- and lifespan in the fish. Additional markers of cellular senescence need to be employed to better assess the senescent cell burden in *N. furzeri* so as to explain the difference in lifespan between the GRZ and MZM strains. Assessing senescent cell burden in different tissues is also import as not all GRZ tissues exhibit the same level of sa *β* gal staining (Schofer et al. 2024 ).

A single bout of fasting improves the healthspan in *N. furzeri* GRZ. SC burden, as assessed by sa *β* gal activity, does not necessarily correspond with lifespan between strains and sexes but does within the male sex of a particular strain. The visible aging of the MZM fish in the absence of a detectable increase in supposed sa *β* gal activity calls into question whether sa *β* gal activity really is senescence-associated in these fish. This indicates that the genetic background of the *N. furzeri* strain is critical to the aging process, and different strains’ aging progression needs to be characterized before comparing results of one study using a particular strain is compared with a study using a different strain. We confirm that intermittent fasting improves health and median lifespan in another model organism.

## Methods

**Animal care and procedures**

Fish of the GRZ and MZM lines were bred and raised in our facility using a recirculating system with UV filter and filled with reconstituted RO water (two tablespoons of Reef Crystals by Instant Ocean) set to 25 *^◦^* C (Polacik et al. 2016). Adult fish were fed Xtreme Aquatic Nano from four-weeks of age. Fry were raised on freshly hatched *Artemia* nauplii. A 5% water change was performed each day. Fish were housed in groups from the same hatch with up to 12 fish per 20 L aquarium (41 *×* 21 *×* 26.5 cm). Plastic plants were provided for behavioral enrichment and for fish to seek shelter from conspecifics (Thore et al. 2020). Both strains originate from the fish used in Pisa to generate the data for Terzibasi et al. (2008). Experiments were conducted under the oversight of the Institutional Animal Care and Use Committee (protocol P240601).

GRZ fish were sampled at 42, 58 and 85 days of age; and MZM fish were sampled at 43, 57 and 141 days of age. Fish were euthanized by MS222 overdose and immersed in ice-water, after which tissue samples were obtained for histological analysis. Fish of the GRZ strain were fasted for 14 days from 42 weeks of age during which they received no food. The fish were separated by size and sex to avoid cannibalism.

**Histology**

Tail portions were fixed in LacZ buffer (0.2% glutaraldehyde, 2 mM MgCl _2_ in PBS) overnight before being decalcified in 10% EDTA with 2 mM MgCl _2_ for 24 hours. Tissues were then processed from 15 to 50% sucrose with an overnight incubation of 50% sucrose-50% OCT solution before impregnation in OCT (Dimri et al. 1995). All steps were at 4 *^◦^* C. 8 *µ* m sections were cut and stored at -80 *^◦^* C until use.

Sections were post-fixed in ice-cold 100% acetone for 10 minutes (Shimada et al. 2012; Komatsu et al. 2012) before proceeding to staining. Tissue was circled with a pap pen and placed in a humidified chamber. Fresh staining solution was prepared before staining (2 mM MgCl _2_ , 5 mM Potassium ferricyanide, 5 mM potassium ferrocyanide, 2 mg/mL X-gal in citrate-phosphate buffer) (Itahana et al. 2007). The pH was set to pH 6.0 following Schöfer et al. (2024). The X-gal was first dissolved in dimethylformamide before dilution into the staining solution just before use. 100 *µ* L of staining solution was added per tissue section and the tissues incubated at 37 *^◦^* C for two hours (GRZ) or four hours (MZM) where after the sections were washed and cover-slipped.

Tissue sections were observed and photographed using a Evos M7000 microscope using the 40 *×* objective.

Cells that developed sa *β* gal activity were counted in ImageJ by eye (Stanley, 2004). An area of skin tissue was marked in ImageJ to determine the pixel area and the number of cells within the marked area counted. Pixel area was normalized to mm ^2^ using the scale bar attached to image by Evos (not shown). Multiple sections per fish were examined. The sa *β* gal positive cell density varies largely between selected areas. The counting of sections and tissue areas continued until average of the counts per mm ^2^ was within 5% of the median count so as to prevent under-sampling skewing the data set.

Images were manipulated in Gimp 2.10.36 (GIMP Development Team, 2024) to correct for background.

**Statistics**

Data was manipulated in Excel. All statistical analyses were performed in R 4.3.3.

The nph, survival, ggplot2 and survminer packages were used for survival analysis and to generate survival curves. Kaplan–Meier analysis was used to generate survival and cumulative hazard plots. The Maximum combination log-rank test (MaxCombo) was used (with Bonferonni correction) for survival analysis. This test avoids assumptions of proportional hazards that would be violated with early or late effects but retains the statistical power of a LogRank test (Lin et al. 2020).

The car, dplyr, emmeans, flextable, ggplot2, ggpubr, officer, rstatix and tidyr packages were used for statistical analysis of the sa *β* gal density. The Shapiro test was used to test for normality and the Levene test was used to test for equal variance. ANOVA and two-way-ANOVA tests were performed using the median density per fish with Tukey post-hoc tests (with Bonferonni correction). Pearson’s correlation coefficient was calculated. Cohen’s d was used to calculate effect size of pair-wise comparisons and *η* ^2^ effect sizes were calculated for ANOVAs.
